# Belimumab or anifrolumab for systemic lupus erythematosus? A risk-benefit assessment

**DOI:** 10.3389/fimmu.2022.980079

**Published:** 2022-08-31

**Authors:** Kyriakos A. Kirou, Maria Dall`Era, Cynthia Aranow, Hans-Joachim Anders

**Affiliations:** ^1^ Department of Medicine, Hospital for Special Surgery and Weill Cornell Medical College, New York, NY, United States; ^2^ Division of Rheumatology, Department of Medicine, University of California, San Francisco, San Francisco, CA, United States; ^3^ Institute of Molecular Medicine, Feinstein Institute for Medical Research, Manhasset, NY, United States; ^4^ Department of Medicine IV, University Hospital of the Ludwig Maximilian University, Munich, Germany

**Keywords:** glucocorticoids, viral infection, belimumab, anifrolumab, systemic lupus

## Abstract

Treatment of systemic lupus erythematosus (SLE) currently employs agents with relatively unselective immunosuppressive properties. However, two target-specific biological drugs have been approved: belimumab (anti-B-cell-activating factor/BAFF) and anifrolumab (anti-interferon alpha receptor-1/IFNAR1). Here, we performed a comparative risk-benefit assessment for both drugs based on the role of BAFF and IFNAR1 in host defense and the pathogenesis of SLE and by considering the available data on safety and efficacy. Due to differences in target expression sites, anti-IFNAR1, but not anti-BAFF, might elicit organ-specific effects, consistent with clinical efficacy data. The IFNAR1 is specifically involved in innate and adaptive antiviral immunity in most cells of the body. Consistent with this observation, the available safety data obtained from patients negatively selected for LN and neuropsychiatric SLE, primary immunodeficiencies, splenectomy and chronic HIV, HBV, HCV infections suggest an increased risk for some viral infections such as varicella zoster and perhaps influenza. In contrast, BAFF is mainly involved in adaptive immune responses in lymphoid tissues, thus anti-BAFF therapy modulates SLE activity and prevents SLE flares without interfering with local innate host defense mechanisms and should only marginally affect immune memory to previous pathogen exposures consistent with the available safety data from SLE patients without chronic HIV, HBV or HCV infections. When using belimumab and anifrolumab, careful patient stratification and specific precautions may minimize risks and maximize beneficial treatment effects for patients with SLE.

## Introduction

Systemic lupus erythematosus (SLE) is a chronic systemic autoimmune disorder presenting with a wide spectrum of clinical manifestations that relate either to systemic inflammation or to autoimmune tissue inflammation and injury (1). Uncontrolled disease activity leads to accumulating tissue injury-related disability and potentially to organ failure (2). Immunosuppressive agents (ISA) can often suppress the aberrant immune response and limit tissue injury. However, drug toxicity remains an important concern (1–3). All currently available drugs for SLE are associated with adverse effects (**Table 1**); thus, developing more specific agents with better safety profiles remains a critical unmet medical need in SLE (3).

**Table 1:** Severe adverse effects of drugs in use for the treatment of SLE.

Target	Drug	Infections	Metabolic	Others
Alkylating agent	CYC	Bacterial, viral, fungi parasites	Anorexia, Liver injury	Cytopenia, nausea, cystitis, bladder cancer, hematologic malignancy, azoospermia, ovarian failure, teratogenicity, alopecia, cardiomyopathy, mucositis
Calcineurin	CyA, Tac	Bacterial, viral, fungal	Diabetes, Hyperlipidemia	Hypertrichosis, tremor, GI symptoms
CD20+ B cells	Rituximab Obinutuzumab	Bacterial, viral	-	Hypersensitivity, hypogamma- globulinemia
Dihydrofolate reductase	Methotrexate	Bacterial, viral (VZV), fungi, parasites	Liver injury	Cytopenia, lung toxicity, hypersensitivity, GI symptoms, alopecia, malignancy
Glucocorticoid receptor	Glucocorticoids	Bacterial, viral, fungi parasites	Weight gain, hyperglycemia, hyperlipidemia, Cushing syndrome hypervolemia	Adrenal insufficiency, hypertension, cataract, glaucoma, osteonecrosis, osteoporosis, myopathy, mood disorders, insomnia, peptic ulcer, acne, skin thinning/ecchymoses, and other
Inosine mono- phosphate dehydrogenase	MMF	Bacterial, viral, fungi parasites	Hyperuricemia	Cytopenia, Diarrhea, teratogenicity, skin rash, hypogamma- globulinemia, malignancy
Lysosomal pH	Chloroquine Hydroxychloroquine	-	Anorexia	Retinopathy, nausea, QTc interval prolongation, cardiomyopathy, myopathy, allergic skin rash, skin hyperpigmentation, methemoglobinemia
Purine-related enzymes	Azathioprine	Bacterial, viral, fungi, parasites	Liver injury	Cytopenia, GI symptoms, alopecia, malignancy

CYC, cyclophosphamide; MMF, mycophenolate mofetil; GI, gastrointestinal; CyA, cyclosporin A; Tac, tacrolimus; BAFF, B cell activating factor; IFNAR, interferon-α/β receptor; VZV, varicella zoster virus.

To date, the U.S. Food and Drug Administration has approved two biological drugs for SLE: the anti-B-cell-activating factor IgG belimumab (anti-BAFF) and the anti-interferon alpha-receptor 1 IgG anifrolumab (anti-IFNAR1). The FDA approved belimumab for the treatment of moderate-severe lupus in patients over 18 years old in 2011, patients with systemic lupus over 5 years old in 2019, and patients with lupus nephritis in 2020. Hence, a considerable amount of efficacy and safety data has accumulated (4). In 2021, the FDA approval of anifrolumab sparked the discussion as to how best to implement this new drug option into the current treatment landscape of SLE (5).

Because head-to-head comparisons between drugs remain scarce, specific recommendations on the preferential use of drugs remain difficult (6). In this article, we discuss the potential risks and benefits of anti-BAFF and anti-IFNAR1 based on their different roles in host defense and the pathogenesis of SLE. We further elaborate on how the ongoing pandemic of coronavirus disease (COVID)-19 may affect risk-benefit assessments for belimumab and anifrolumab.

## Different roles of BAFF and the IFNAR1 in host defense

**Figure 1:**
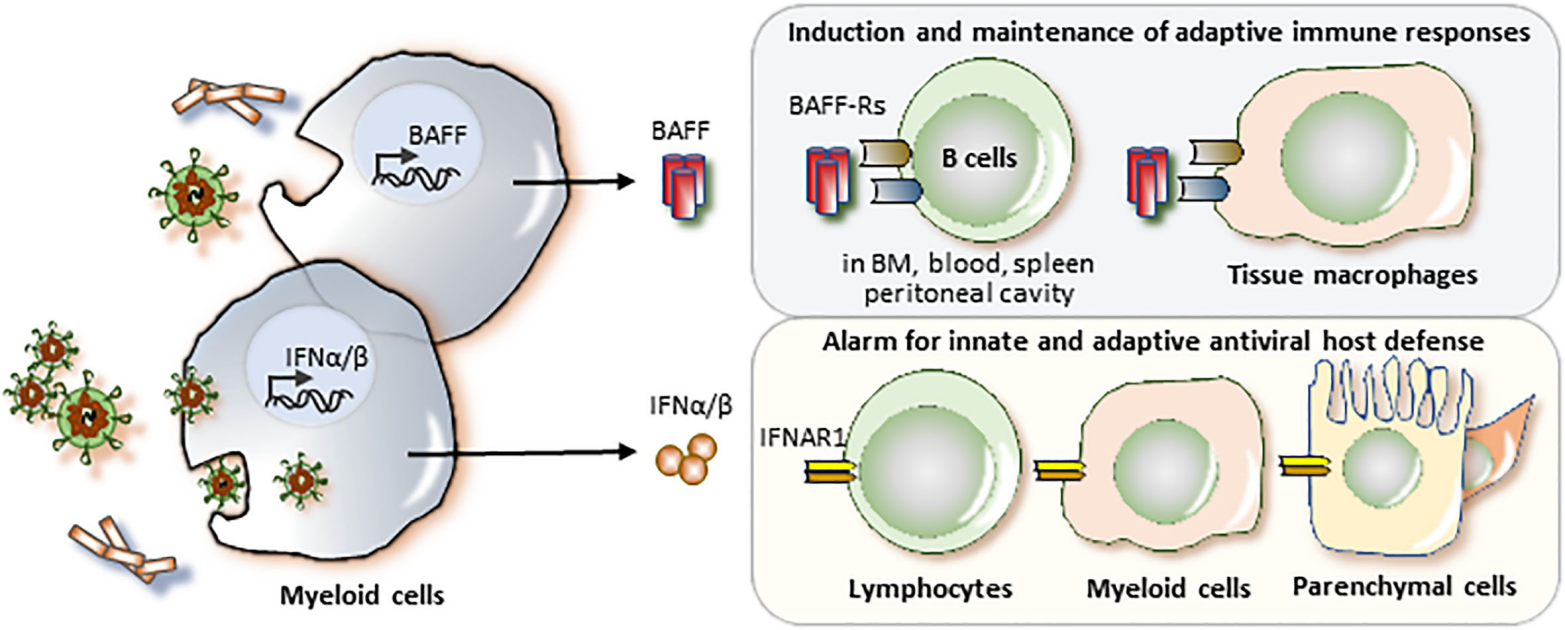
BAFF and the IFNAR1 in host defense. All classes of pathogens release pathogen-associated molecular patterns (PAMP) that can activate pattern recognition receptors in myeloid cells to upregulate the expression and release of B cell activating factor (BAFF) and/or interferon-alpha and -beta (IFNα/β). Viruses are particularly potent in inducing IFNα/β as their viral nucleic acid activate specific nucleic acid sensors in intracellullar compartments such as phagolysosomes inside the cytosol. BAFF mediates its biological effects *via* the BAFF receptor (BAFF-R) on T1 B cells in the bone marrow, blood, and spleen, on B1B cells in the peritoneal cavity, and on follicular B cells and marginal zone B cells in the spleen. BAFF has a specific role in the induction and maintenance of adaptive immune responses. In addition, tissue macrophages express BAFF-R and IFNAR1 and BAFF and IFNAR1 ligation both induce a M1 proinflammatory macrophage phenotype, an innate mechanism of host defense. In addition, nearly all cells of the body express the IFNAR1. Activation of the IFNAR1 activates immune and tissue cells to induce several hundred genes important in antiviral host defense.

BAFF and the IFNAR1 are both elements of the immune system and, thus, their primary function in human physiology relates to host defense (7, 8). Only the IFNAR1 is broadly expressed as part of the tissue`s innate immune system (**Figure 1**) (8). The innate immune system supports host defense in all plants and animals (9) (10) and all immune cells express the IFNAR1 to mediate the numerous biological effects of the type I IFNs (IFN-I, **Figure 1**) (11). The IFNAR1 is a key element of the danger alert system that converts local danger recognition into systemic inflammation and induction of danger resilience at sites distant from pathogen entry (8). In this process, IFNAR1 signaling is linked to the sensing of viral components (12). In tissue macrophages, type I IFNs induce a pro-inflammatory M1 phenotype (13). In addition, IFNAR1 signaling induces the maturation of antigen-presenting cells and, therefore, triggers the initiation and persistence of adaptive immunity (8, 14). In addition, the vast majority of non-immune cells express the IFNAR1 to activate innate and adaptive host defense mechanisms.

In contrast, the expression of BAFF receptors is restricted to certain maturation stages of B cells, dendritic cells and tissue macrophages (**Figure 1**) (7). BAFF supports the maturation, differentiation, and survival of B-lymphocytes (7), which serve two central roles in the adaptive immune system: (i) B cells are antigen-presenting cells and therefore key initiators and drivers of antigen-specific T cell immunity (15, 16). and (ii) B cells are effector cells of the adaptive immune system and produce immunoglobulins as they undergo maturation into plasma cells (17). Both functions of B cells are critical for the development of long-lasting antigen-specific immune memory (18, 19), which limits the severity of recurrent infections and accounts for relapsing chronic autoimmune diseases such as SLE (16, 20). Thus, in contrast to the IFNAR1, targeting BAFF is associated with greater selectivity.

*BAFF and IFNAR1 in anti-bacterial host defense.* Antibacterial host defense involves all aspects of innate and adaptive immunity. Toll-like and other pathogen recognition receptors detect a wide spectrum of bacterial components and trigger a diverse range of cytotoxic mechanisms (9). The system is highly redundant, leaving many specific genetic defects without clinical consequences (21). Indeed, severe bacterial infections relate more to profound and diffuse immunodeficiencies, such as in the setting of hereditary or acquired immunoglobulin deficiencies, treatment with glucocorticoids (GC), nonselective ISA or advanced organ failure (heart, kidney, liver) (21). Bacterial products activate BAFF secretion from myeloid cells; hence, during infection, BAFF levels increase in biological fluids. BAFF deficiency increases the susceptibility of mice to certain bacterial strains but BAFF does not seem to have a non-redundant role in innate host defense against bacteria in humans (22). In contrast, BAFF is necessary for priming adaptive immunity during *de novo* exposure to a new bacterial pathogen. IFN-I and the IFNAR1 partially contribute to antibacterial host defense by sensing bacterial CpG-DNA and potentially de-methylated nucleic acids released from infected cells (23).

*BAFF and IFNAR1 in antiviral host defense*. The sensing and priming of antiviral immunity differ significantly from antibacterial immunity. Viral nucleic acids play an important role in sensing viral infections and involve a set of intracellular nucleic acid sensors that all trigger the secretion of type I/III IFNs as the central cytokine signals of antiviral immunity. IFN-I activate the IFNAR1, which not only induces the release of nucleases and viral transcription inhibitors to limit viral spreading throughout the body but results also in systemic symptoms such as malaise, fatigue, fever, arthralgia or myalgia. In addition, IFNAR1 activation-related BAFF release from immune cells supports antigen-presentation and the expansion of viral antigen-specific B cells. This applies especially for *de novo* exposures to previously unknown viruses. Thus, the risk for viral infections could be higher with therapeutic IFNAR1 inhibition compared to therapeutic BAFF inhibition. BAFF released from neutrophils and dendritic cells contributes to antiviral immunity (24), but accelerates gamma herpesvirus infection (25). Both therapeutics might affect the priming of immune memory to novel viruses or vaccines.

*BAFF in host defense to plasmodia.* In human malaria infection, BAFF levels increase and IgD (–)CD38(low)CD21(-)CD27(-) atypical B cells temporarily expand (26). An interesting line of evidence suggests that BAFF plays an important role in the host defense to plasmodia falciparum. The population of Sardinia has an unusually high prevalence of SLE in association with a genetic BAFF variant, referred to as “BAFF-var” (27). BAFF-var is an insertion-deletion, which ultimately increases BAFF levels likely accounting for the unique high prevalence of SLE in Sardinia compared to the rest of Europe (27). An extraordinarily high prevalence of certain genetic variants frequently relates to distinct infectious selection pressures such as apolipoprotein-1 variants that confer some protection from severe trypanosomiasis or hemoglobin variants that protect from severe malaria (28, 29). Indeed, malaria was endemic in Sardinia until the 1950s, and BAFF-var-related BAFF overexpression enhances the mucosal immune response against plasmodia falciparum (30). Hence, therapeutic BAFF inhibition might increase the risk for parasite infections such as malaria, a specific exposure risk, underrepresented in the recent clinical trials.

## Infectious complications with anti-BAFF and anti-IFNAR1

*Anti-BAFF.* Since its initial approval by the U.S. Food and Drug administration (FDA) in March of 2011, belimumab has consistently demonstrated an overall reassuring safety profile in comparison to standard of care (SOC) therapy in both adult and pediatric SLE patients (**Table 2**). One study analyzed pooled data from 1458 adult participants treated with placebo or belimumab at doses of 1 mg/kg, 4 mg/kg, or 10 mg/kg on background standard of care therapy from the phase II, BLISS-52 phase III, and BLISS-76 phase III trials (41). The trial durations ranged from 52-76 weeks. All treatment groups had similar rates of overall adverse events (AEs), treatment-related AEs, serious AEs, and AEs resulting in treatment discontinuation. The rates of infectious AEs were balanced among the treatment groups. Of note, three opportunistic infections occurred in two participants in the belimumab groups. One participant in the 10 mg/kg group developed acinetobacter bacteremia and later disseminated cytomegalovirus infection (both resolved) and one participant in the 1 mg/kg group developed Acinetobacter iwolfii pneumonia on study day 1 (resolved). Rates of serious psychiatric disorder and depression were higher in the belimumab groups and there were two completed suicides, one each in the 1mg/kg and 10mg/kg groups.

**Table 2:** Infection-related safety data of anti-BAFF and anti-IFNAR1 from large RCTs.

Drug	(Likely) bacterial infection	(Likely) viral infection	All infection	Seriousinfection	Ref.
**Anti-BAFF**					
BLISS-52	UTI 16 vs. 16% Sinusitis 11 vs. 10% Bronchitis 12 vs. 8%	Upper RTI 20 vs. 21% Nasopharyngitis 16 vs. 9%	64 vs. 67%	6 vs. 4%	(31)
BLISS-76	UTI 16 vs. 16% Sinusitis 11 vs. 10% Bronchitis 12 vs. 8%	Upper RTI 20 vs. 21% Nasopharyngitis 16 vs. 9%	74 vs. 69%.	7 vs. 6%.	(32)
BLISS-SC	Sepsis 1 vs. 1%	Herpes zoster 3 vs. 4%	55 vs. 57%.	4 vs. 5%	(33)
BEL113750	Upper RTI 3 vs. 6% UTI 4 vs 1%	Upper RTI 6 vs. 7% Nasopharyngitis 12 vs. 11% Herpes zoster 5 vs. 5%	n.r.	5 vs. 6%	(34)
PLUTO	Pneumonia 0 vs. 3% Abscess 4 vs. 0% Epiglottitis 0 vs. 3%	Herpes zoster 2 vs. 3% Influenza 0 vs. 3% Hepatitis A 0 vs. 3%	57 vs. 70%.	8 vs. 13%.	(35)
BLISS-LN	Bronchitis 5 vs. 4% Pneumonie 1 vs.2% UTI 7 vs. 6%	Upper RTI 12 vs. 11% Nasopharyngitis 4 vs. 4% Herpes zoster 6 vs. 4%	13 vs. 15%.	7 vs. 8%	(36)
**Anti-IFNAR1**					
MUSE	Bronchitis 4 vs. 4% Sinusitis 6 vs. 3%	Upper RTI 13 vs. 10% Influenza 6 vs. 2% Herpes zoster 5 vs. 2%	n.r.	n.s.	(37)
TULIP 1	UTI 12 vs. 15% Bronchitis 9 vs. 5% Pneumonia 2 vs. 1%	Nasopharyngitis 20 vs. 12% Upper RTI 12 vs. 10% Herpes zoster 6 vs. 2%	n.r.	6 vs 5%	(38)
TULIP 2	Bronchitis 12 vs. 4% UTI 11 vs 14% Sinusitis 7 vs. 5% Pneumonia 4 vs. 2% Tuberculosis 2 vs. 0%	Upper RTI 22 vs. 10% Nasopharyngitis 15 vs. 11% Herpes zoster 7 vs. 1% Influenza 2 vs. 3% Gastroenteritis 2 vs. 4%	n.r.	n.r.	(39)
TULIP-LN	UTI 17 vs. 10% Bronchitis 12 vs. 12%	Upper RTI 16 vs. 16% Nasopharyngitis 16 vs. 18% Herpes zoster 17 vs. 8% Influenza 8 vs. 2% Oral herpes 6 vs. 4% Herpes simplex 5 vs. 4%	n.r.	2 vs. 6%	(40)

The percentages represent study drug versus (vs.) placebo. Belimumab trial data are shown for the 10 mg/kg dose. Anifrolumab trial data are shown for the 300 mg/kg dose, except for TULIP-LN, which combined basic and intense regimen. RCTs, randomized controlled trials; URTI, upper respiratory tract infection; Sinus., Sinusitis; UTI, urinary tract infection; Nph, nasopharyngitis; CAP, community acquired pneumonia; n.r., not reported.

In terms of laboratory parameters, more participants in the belimumab groups experienced reductions in IgG, IgM, and IgA levels below the lower limit of normal. However, there was not an observed association between infectious AEs and reduction in immunoglobulin levels.

The longest study of belimumab completed to date is the open-label continuation study of the phase II, double-blind trial (42). All participants received the licensed dose of belimumab 10mg/kg IV every 4 weeks in addition to standard background therapy. 298/476 (63%) entered the continuation study after completing the phase II trial. 96 (32%) participants remained in the study until the end at year 13. Over the course of the study, 44 (14.9%) discontinued therapy or withdrew from the study because of an AE. The rates of serious infections were stable over the course of the study. There were no cases of progressive multifocal leukoencephalopathy, although several such cases were reported in patients treated with belimumab outside clinical trials (43).

BASE was a 52- week multicenter, double-blind, randomized, placebo-controlled phase IV trial required by both the FDA and the European Medicines Agency to investigate the rates of all-cause mortality and adverse events of special interest in belimumab versus placebo on background standard of care (44). The as-treated population included 2002 belimumab-treated participants and 2001 placebo-treated participants. The incidence of all-cause mortality was similar between the two groups, 0.5% and 0.4% in the belimumab and placebo groups, respectively. Serious infections occurred in 3.7% in the belimumab group and 4.1% in the placebo group. There was no significant difference in the frequency of opportunistic infections, infections of special interest, and malignancies between the two groups. More belimumab-treated participants experienced serious depression than those treated with placebo (0.35% versus 0.05%).

Lastly, in the PLUTO trial of participants with childhood SLE, belimumab was well-tolerated and the safety profile was similar to the experience in adult SLE patients described above (35). Together, even long-term inhibition of BAFF does not seem to be associated with significant increases in the rates of infections, although exposures to plasmodium falciparum or other important (sub-)tropical parasites may have been rare in these studies. Interestingly, COVID-19 outcomes have been more favorable in the small subset of patients with SLE treated with belimumab (45, 46).

*Anti-IFNAR1.* Anifrolumab was approved by the FDA as the 2^nd^ biologic available for the management of moderate-severe systemic lupus in patients over 18 years old in 2021. Anifrolumab was approved based on data from three randomized double-blind, placebo controlled, 52-week clinical trials: the MUSE phase II and the phase III TULIP 1 and 2 clinical trials (37–39). These trials excluded patients with LN and neuropsychiatric SLE, primary immunodeficiencies, splenectomy and positive serologies for HIV, HBV, HCV. Patients with history of severe herpes [disseminated HZ involving ≥3 dermatomes, herpes encephalitis, ophthalmic herpes, or recurrent HZ (2 episodes within 2 years)] were also excluded. In addition, patients with a history of recent severe opportunistic infections and recent chronic infections (i.e., osteomyelitis; bronchiectasis) were excluded. Pooled safety data from all 3 trials were analyzed by Tummala et al. (47). The safety analysis focused on the comparison of 459 patients that received the FDA approved 300 mg dose with 466 patients that had placebo. The anifrolumab 300 mg and placebo groups were well balanced regarding demographics, comorbidities, treatment with GC, antimalarials, and ISA. More patients in the anifrolumab 300 mg group (87%) had ≥1 AE compared to those on placebo (79%). AEs more common in the anifrolumab 300 mg group than in placebo were nasopharyngitis (16% vs 9%), URTI (16% vs 10%), bronchitis (10% vs 4%), and HZ (6.1% vs 1.3%). Most were mild or moderate. Severe AE (SAE) occurred in 11.8% and 16.7% receiving anifrolumab 300 vs placebo. Among SAE, infection rates were 22 (4.8%) versus 26 (5.6%). There were 2 deaths due to pneumonia in the anifrolumab 300 mg group and 1 in the placebo group due to encephalitis. There was also one death in the anifrolumab 1000 mg group due to acute colitis and macrophage activation syndrome. There were two severe opportunistic infections: one on anifrolumab 300 with mycobacterium complex infection (treatment was discontinued), and one on placebo with cryptococcal meningitis. There were no active tuberculosis cases but four (0.9%) and one (0.2%) patient, respectively, with latent tuberculosis. HZ occurred in 28 patients (6.1%) in the anifrolumab 300 group and 6 patients on placebo (1.3%). Only two HZ cases (both on anifrolumab 300) were SAEs: 1 in MUSE trial had transverse myelitis with positive PCR for the virus in the cerebrospinal fluid but no cutaneous symptoms. The patient discontinued treatment and fully recovered with antivirals and high dose GC. All cases received antiviral treatment (except 2 in the placebo group) and all resolved. In the TULIP studies (dermatome involvement data were available), 4/28 cases were disseminated: 3/23 in the anifrolumab 300 and 1/5 in the placebo group. Of note, the rate of HZ was higher in the anifrolumab 300 group who received ISA (n=17, 9.8%) than those who did not (n=6, 3.2%). In the MUSE trial, the HZ rate was higher for the anifrolumab 1000 group (9.5%) compared to anifrolumab 300 (5.1%) and placebo (2%) groups (37).

The TULIP-LN, phase II double-blind randomized trial of anifrolumab in patients with active class III/IV lupus nephritis (LN) was recently published (40). In this study 147 patients were randomized to receive the monthly anifrolumab basic regimen (BR, 300 mg, n=45), an intensified anifrolumab regimen (IR, 900 mg X3 followed by 300 mg, n=51), or placebo (n=49), for 52 weeks. Eligible patients could enter a 2^nd^ year of treatment. Only the first-year data were published. AE occurred in 90 (94%) of patients in the combined anifrolumab group compared to 44 (90%) in placebo, but were only mild/moderate in intensity,. SAE occurred in 20% of anifrolumab and 16% of placebo groups. AEs more common in anifrolumab than placebo included HZ (17% vs. 8%), influenza (8% vs. 2%), and UTI (17% vs. 10%). Of the 16 HZ cases in the combined anifrolumab group, 6 were serious but only cutaneous and resolved with antiviral treatment.

The longest anifrolumab safety data are available from the MUSE open-label 3-year extension study (48). Of the 246 patients who completed the MUSE trial, 218 (88.6%) enrolled in this study and 139 (63.8%) completed 3 years of treatment. Frequency and patterns of SAE and AESI over 3 years were consistent with those reported for one year in patients on anifrolumab in the parent study. HZ occurred in 11 patients (5%; 2 were disseminated, but not serious) in 3 years compared to 15 (7.4%) in the parent trial. There were 6 patients (2.8%) with latent tuberculosis compared to 2 (1%) in MUSE. A further placebo-controlled long-term observation extension study of patients enrolled in TULIP1 and 2 trials is ongoing.

While these studies suggest that anifrolumab does not increase serious adverse events in SLE, there was an increase in the frequency of upper respiratory tract infections, nasopharyngeal, bronchitis and HZ (49). Limitations in these data include the relatively short follow-up available and the selection of patients at lower risk for HZ and other infections. In addition, the numerically higher incidence of latent tuberculosis and influenza infections in some of these trials needs further study.

## BAFF and IFNAR1 in the pathogenesis of SLE disease activity

BAFF and IFNAR1 are both involved in the early stages of the pathogenesis of SLE. Because the diagnosis of SLE is founded on the presence of antinuclear antibodies (50), loss-of-tolerance and priming of autoreactive lymphocyte clones has already occurred at the time of diagnosis. Importantly, the presence of antinuclear antibodies is usually long-lasting, implying persistent memory T cells and long-lived plasma cells (51). To address these factors, we have chosen to discuss the role of BAFF and the IFNAR1 in relation to the level of SLE disease activity.

**Figure 2:**
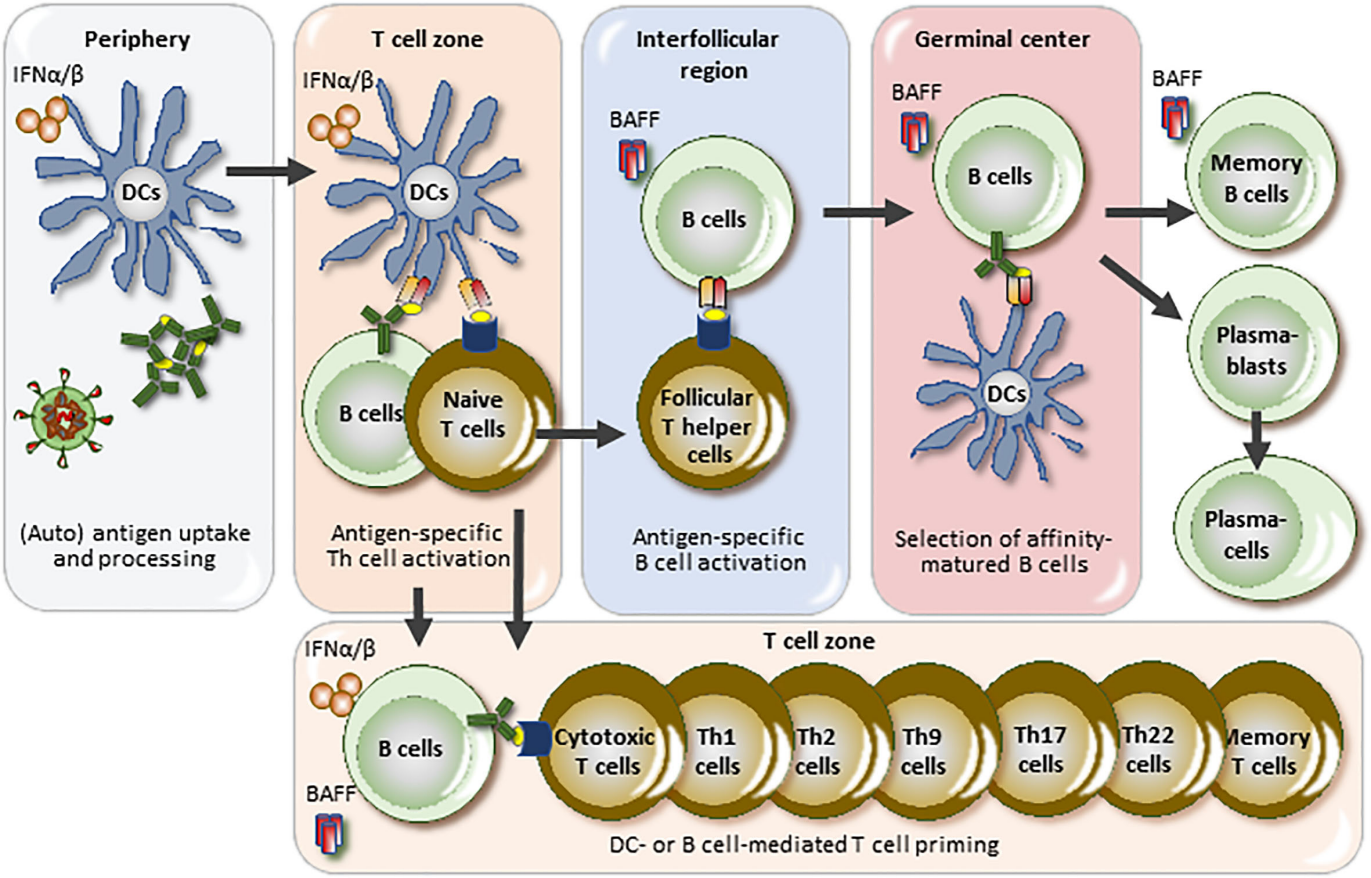
BAFF and IFNAR1 in the pathogenesis and disease activity of SLE. Lupus autoantigens stimulate antigen-presenting cells to secrete interferon-alpha and -beta (IFNα/β) just like viral particles, a process that triggers an antiviral-like adaptive immune response against lupus autoantigens. Lupus activity largely depends on the size and activity of the autoreactive T and B cell clones and IFNAR1 signaling is centrally involved in clone size regulation triggered by extracellular lupus autoantigens but not necessarily by drug- or bacterial infection-related lupus flares. B cell-activating factor (BAFF) is specifically involved in B cell maturation towards plasma cells but may also endorse B cell-mediated T cell priming. DC, dendritic cell.

*BAFF and IFNAR1 in SLE disease activity.* The central element of the pathogenesis of SLE is the loss of tolerance to chromatin (1). Monogenic forms of SLE provide clues for key triggers of this process such as the hereditary overproduction of IFN-I, hereditary defects in apoptosis, opsonins or chromatin clearance defects all promoting the priming of anti-chromatin immunity (52). In this context, IFNAR1 signaling is the central element of monogenic interferonopathy-related SLE and critically involved in the innate sensing of (viral particle-like) self-chromatin as well as the priming of adaptive anti-chromatin immunity (**Figure 2**) (53). For example, Toll-like receptor 7, a viral RNA recognition receptor sensing also lupus autoantigens, is a key driver of murine and human SLE and mediates the resistance of SLE activity to, e.g., glucocorticoids (54–59). Thus, the IFNAR1 mediates SLE activity whenever chromatin release into the extracellular space boosts anti-chromatin immunity, e.g., cell death related to sunburns, trauma or NET release during infection (14). In addition, viral infection-related flares of SLE likely involve the IFNAR1, while infections related to other microbes likely trigger disease flares *via* alternative signaling pathways not necessarily involving the IFNAR1, e.g., Toll-like receptors-2, -4, and -5 for gram-negative and gram-positive bacteria (60). However, there are not yet data demonstrating that blocking the IFNAR1 with anifrolumab reduces SLE flare rates. In contrast, belimumab attenuates the frequency and severity of SLE flares (61, 62), even if the role of BAFF is limited to the adaptive immune system. Obviously, BAFF-related B cell functions including antigen-presentation, production of autoantibodies (**Figure 2**), and circulating immune complexes predominate as drivers of human SLE activity, while the role of the innate immune system had been demonstrated by animal studies (63, 64). Thus, flares of SLE activity involve BAFF-dependent B cell functions such as autoantigen-presentation, expansion of autoreactive T and B cell clones (65).

*BAFF and IFNAR1 in specific organ manifestations of SLE*. SLE is a systemic disorder with much of its pathophysiology localizing to the lymphoid organs, i.e., spleen and lymph nodes (1). Thus, organ-specific features may be associated with factors acting in the periphery, such as the IFNAR1, but not to factors acting preferentially in the lymphoid organs such as BAFF (**Figure 2**). Thus, certain organ manifestations in SLE may relate to local IFNAR1 activation, e.g., in the skin, the synovium, central nervous system, kidneys and blood vessels, either due to infection, UV light (in skin), or plasmacytoid dendritic cell migration to these sites. Evidence for IFNAR1 activation in skin (66, 67), CNS (68), synovium (69), kidneys (67), and endothelial cells has been described (70). In addition, high systemic type I IFN activity associates with active class III/IV LN (71).

Viral infections are frequent in the respiratory tract, while persistent viruses reside in the skin, the nervous system, the liver and the immune system (72). Indeed, a comparative single cell transcriptome analysis found strong IFN signaling in lupus skin (67).

In summary, while BAFF should be involved in all forms of flaring SLE activity, the IFNAR1 could be specifically involved in flares triggered by viral infections and release of endogenous chromatin antigens (73, 74). The expression of the IFNAR1 in peripheral tissues may involve IFNAR1 differently in the various SLE manifestations, which is less likely for BAFF.

## Efficacy data of anti-BAFF and anti-IFNAR1 in SLE

*Anti-BAFF.* Belimumab received FDA approval in 2011 for treatment of moderate-severe systemic lupus in patients over 18 years old based upon efficacy demonstrated in BLISS-52 (n=577) and BLISS-76 (n=548) (31, 32). In BLISS-52, subjects were randomized to receive standard of care plus 1 mg/kg, 10 mg/kg belimumab or placebo every 4 weeks with a loading dose administered at week 2. After 52 weeks, subjects receiving belimumab 10 mg/kg were statistically more likely to achieve an SRI-4 response compared to those receiving placebo (58% vs. 44%, p<0.001). Similarly, in BLISS-72, subjects randomized to intravenous belimumab 10 mg/kg achieved an SRI-4 response statistically more frequently than those receiving placebo infusions (43% vs. 34%, p=0.021). Evaluation of a subcutaneous route of administration (200 mg/week) in an 839 subject trial showed achievement of an SRI-4 in 61% of subjects receiving belimumab compared to 48% in those receiving placebo (p=0.0006) and led to the approval of subcutaneous belimumab in 2017 for active autoantibody positive SLE. A subsequent study in Asia (n=707) confirmed the superior efficacy of intravenous belimumab compared to placebo (SRI-4 54.3 vs 40.1 p<0.00001) in this population. In 2019, Belimumab was approved for use by pediatric patients with SLE following an international study (PLUTO) in which 93 subjects, aged 5-17 years, were randomized to receive belimumab (10mg/kg) or placebo. Numerically greater numbers of subjects receiving belimumab achieved a SRI-4 (52.8% vs 43.6%; OR 1.49 (95% CI 0.64 to 3.46)) (35). These studies have consistently shown a clinical effect differentiating belimumab treatment from placebo after 16 weeks of therapy, sustained through the 52-week endpoint. Additionally, compared to placebo, patients treated with belimumab have lower frequencies of severe flare and prolonged median times to flare. GC tapering was not mandatory and left to the investigator’s discretion in these studies.

In BLISS-52, patients receiving belimumab were significantly more likely to achieve a 50% reduction in GC exposure, while trends for decreased GC exposure have been demonstrated in the other studies. *Post-hoc* examination of the pooled BLISS-52 and BLISS-76 studies showed that a greater number of subjects receiving belimumab had a decrease in their GC dose, and a lower number had increases. Furthermore, GC dose was lower in subjects receiving belimumab (75). Additional *post-hoc* studies of the pooled BLISS-52 and BLISS-76 results demonstrated that improvement occurs across multiple domains and is not restricted to a single organ (76). Patients with high disease activity (i.e., a Safety of Estrogens in Lupus National Assessment–Systemic Lupus Erythematosus Disease Activity Index (SELENA-SLEDAI) ≥10], hypocomplementemia, anti-dsDNA positivity and/or use of prednisone at baseline are the most likely individuals to attain a clinical response compared to SOC (77). Subsequently, studies of long-term use of belimumab have demonstrated that its use is associated with a reduction in accrual of damage (78–80).

Belimumab, both intravenous and subcutaneous, received FDA approval for the treatment of active lupus nephritis following the 104-week BLISS-LN study (36). In this Phase III trial, 448 patients with Class III, IV (with or without Class V) or Class V LN, received either intravenous belimumab 10 mg/kg or placebo along with standard of care (cyclophosphamide/azathioprine or mycophenolate mofetil). Patients receiving belimumab were significantly more likely to achieve a primary efficacy renal response (PERR) at week 104. PERR included a urinary protein to creatinine ratio of ≤0.7, an estimated glomerular filtration rate [eGFR], which could not be ≥ 20% below the pre-renal flare value or ≥60 ml per minute per 1.73 m^2^ of body-surface area, and no use of rescue therapy). Complete renal response was defined as a urinary protein to creatinine ratio of <0.5, an eGFR, which could not be ≥10% below the pre-renal flare value or ≥90 ml per minute per 1.73 m^2^ of body-surface area, and no use of rescue therapy. Belimumab and placebo achieved, 43% vs. 32% (p=0.03) and 30% vs. 20% (p=0.02) for PERR and CR, respectively.

*Anti-IFNAR1.* Anifrolumab was approved for the treatment of SLE based on consistent efficacy data from the phase IIb MUSE and phase III TULIP 1 and 2 trials (81). In MUSE, 305 patients were randomized to receive IV anifrolumab 300 mg (n=99), 1000 mg (n=104), or placebo (n=102), every 4 weeks for 48 weeks (37). The primary endpoint was the percentage of patients achieving an SRI-4 at week 24 with concurrent sustained reduction of oral GC to <10 mg/day and not higher than the baseline dose. GC taper was encouraged but not required by the protocol. More patients treated with anifrolumab 300 mg (34.3%) and 1000 mg (28.8%) responded compared to placebo (17.6%). P values were 0.014 and 0.063 respectively for the two experimental groups vs placebo. Improved responses were also seen for SRI-4, and BICLA responses at 52 weeks. The two phase III anifrolumab trials had a similar design, except that TULIP 1 also included a lower anifrolumab dose (150 mg) group. There was a requirement for patients that received baseline GC doses ≥ 10 mg/day to attempt to taper the dose to ≤ 7.5 mg/day from weeks 8-40.

TULIP 1 randomized 457 patients to IV anifrolumab 300 mg (n = 180), 150 mg (n = 93), or placebo (n = 184) every 4 weeks for 48 weeks (38). The primary endpoint was the SRI-4 at week 52 and there was no difference between the two groups. However, after amending the NSAID use responder rules, *post hoc* analysis showed all outcome measures improved, although the primary endpoint was still not significant. Of note, BICLA responses at week 52 were in favor of anifrolumab 300 mg (46%) versus placebo (30%). Pharmacodynamic assessment of IFN-High patients showed neutralization of IFN-I 21 gene panel to a level of 12.6% as early as week 12 and throughout the study period for the anifrolumab 300 mg group but not for the placebo group. The experience from the TULIP-1 study regarding NSAID restriction rules and BICLA vs SRI-4 responses, informed a protocol amendment of the twin study TULIP 2, before that data were unblinded (39). The primary endpoint was changed from SRI-4 to BICLA response at week 52. Flares were defined as ≥ 1 new BILAG-2004 A item or ≥ 2 new BILAG-2004 B items, as compared with the previous visit. TULIP 2 randomized 362 patients to anifrolumab (n=180) or placebo (n=182). The study reached its primary endpoint, as 47.8% patients on anifrolumab compared to 31.5% of the placebo group responded according to BICLA). Regarding key secondary outcomes, this was also true for the IFN-I-High group (48% versus 30.7%; p=0.002), sustained GC reduction (51.5% versus 30.2%; p=0.01), and ≥50% reduction in CLASI at week 12 (49% versus 25%; p=0.04).

The data from the two TULIP trials have been pooled and analyzed (82, 83). There were 360 patients in the anifrolumab 300 group and 366 in the placebo group. Two thirds of the patients were white, 13% AA, 10.5% Asians, and 8.4% other. About 52% were on prednisone ≥10 mg/day at baseline, 70% were on antimalarials, and about 48% on ISA (mycophenolate mofetil ≤ 2g/day, mycophenolic acid ≤ 1.44 g/day, azathioprine ≤ 200 mg/day, methotrexate ≤ 25 mg/week, or mizoribine ≤ 150 mg/day). 70% had SLEDAI-2K≥10, and 59% had ≥1 abnormal serologic markers at baseline (high levels of anti-dsDNA, or low levels of C3 or C4). About 28% had a CLASI-activity ≥10 and 41% had ≥ 6 tender and ≥6 swollen joints. More patients in the anifrolumab 300 mg group achieved BICLA response compared to the placebo group (47.5% vs 30.8; difference 16.6%; nominal p<0.001) (83). Similarly, more patients in the anifrolumab 300 mg group achieved SRI-4 responses (52.2% vs 40.1%), sustained GC taper (50.5% vs 31.8%), ≥50% reduction in CLASI-activity (46% vs 24.9%), ≥50% reduction in active joints (49.4% vs 36.8%) compared to PLB treated patients; all with significant nominal p values. Pooled analysis of the combined TULIP data with emphasis on lupus flares, is also available (82). Most flares were in the mucocutaneous (24.8%) and musculoskeletal (22.5%) systems, and much fewer flares in the renal (6.2%), cardiorespiratory (2.5%) and other systems. The anifrolumab 300 mg group had a lower annualized flare rate (AFR) compared to placebo (0.51 versus 0.67; rate ratio 0.75; P=0.017), prolonged median time to first flare (140 days versus 119 days; hazard ratio 0.70; P = 0.003), and fewer patients with ≥1 flare (33.6% vs 42.9%; difference -9.3%; p=0.009). AFR favored the anifrolumab group for patients with BMI ≤ 28 kg/m^2^, patients on GC ≥10 mg/day, baseline SLEDAI-2K≥10, active baseline serology (≥1 marker), and IFN-I-High patients. Of note, flare rates were similar (~34%) for all placebo-treated patients, but higher for placebo-treated IFN-I High patients (45%). Importantly, among patients who achieved sustained GC reductions more remained flare-free with anifrolumab (40%) versus placebo (17%).

The primary outcome of the TULIP-LN trial was change in 24-hour urinary protein creatinine ratio (UPCR) at week 52 for the combined anifrolumab (BR and IR) groups vs placebo (40). Secondary endpoint was complete renal response (CRR), defined as 24-hour UPCR ≤ 0.7 mg/mg, eGFR≥60 ml/min/1.73 m^2^, or no decrease ≥20% from baseline, no investigational product discontinuation, and no use of restricted medications. There was no difference in the primary endpoint between the combined experimental and placebo groups. However, there were numerical improvements in CRR attained by the IR group versus placebo (45.5% vs 31.1%), as well as in more stringent CRR definitions, and in sustained GC dose reductions (55.6% vs 33.3%). Such improvements were not seen in the BR group vs placebo. Due to proteinuria, serum concentrations were higher in the anifrolumab IR group but suboptimal in the BR group (below those expected in similarly treated non-renal SLE patients) limiting this group’s exposure to anifrolumab and any potential benefit.

Collectively these data confirm the efficacy of anifrolumab on many clinical outcomes, including BICLA and SRI-4, mucocutaneous and musculoskeletal outcomes, lower flare rates, successful GC taper to ≤7.5 mg/day for those on baseline doses ≥ 10 mg/day, increases in C3 levels, as well as in functional measures. Interestingly, the data suggest a preferred beneficial effect in patients with IFN-I pathway activation, patients with active mucocutaneous disease, higher serologic activity, and Asians. Pharmacodynamic neutralization of the IFN-I pathway was consistently seen in the IFN-I High patients treated with anifrolumab 300 mg which corroborates the efficacy findings.

## Risk-benefit assessments for the use of anti-BAFF and anti-IFNAR1 in SLE

Both anti-BAFF and anti-IFNAR1 have demonstrated efficacy in controlling SLE activity. A recent indirect treatment comparison analysis suggested that patients with moderate-to-severe SLE are more likely to achieve an improvement in disease activity with anifrolumab (84), but such types of analysis have to be handled with caution due to the many confounding differences between the respective trials. In addition, such comparisons do not inform about which drug to choose in which patient.

Drug safety profiles largely guide treatment decisions, and indeed, the safety profiles of belimumab and anifrolumab are quite different. More patients treated with belimumab developed serious depression, treatment-emergent suicidality, sponsor-adjudicated serious suicide, or self-injury events (44). On the other hand, severe COVID-19 outcomes in individuals with SLE were favorable with belimumab (45, 46). Unlike anti-BAFF, anti-IFNAR1 significantly increased the rates of certain viral infections such as HZ and perhaps influenza, a finding that connects well with its mechanism-of-action that blocks a key element of antiviral immunity. How anti-IFNAR1 therapy affects the risk of COVID-19 and its complications is not known, but numerous data support that lacking capacity to rapidly induce a IFN-I response is a crucial risk factor for severe COVID-19 (85), although type III IFN also seem to be important (86). Moreover, the presence of autoantibodies against type I IFNs predisposes patients to life-threatening COVID-19 (87). Thus, although we do not have definite data, it remains possible that treatment with anti-IFNAR1 will have a similar effect. In view of these potential infectious risks, full vaccination against HZ and SARS-Cov-2 (avoiding live vaccines) is strongly recommended before starting anti-IFNAR1 therapy for SLE and is probably advisable for any other biological therapy or ISA. Modern antiviral drugs or even passive immunization with SARS-CoV-2 antibodies can protect from severe COVID-19 in patients at risk (88, 89). Caution should be used when considering anti-IFNAR1 treatment in patients with a history of chronic or recurrent infections or at high risk for infections. Prompt identification and treatment of HZ is recommended.

Economic considerations may influence individual patient treatment decisions. We made the deliberate decision not to discuss financial issues in this review. Belimumab and anifrolumab are both biological drugs with relatively higher costs than conventional drugs, and reimbursement rates vary among geographical regions and insurance carriers.

## Summary

The FDA approved belimumab and anifrolumab for the treatment of moderate-severe SLE because both drugs convincingly demonstrated efficacy beyond standard-of-care. Anti-BAFF blocks a key element of the adaptive immune system involved in SLE without interfering with innate host defense, which explains why anti-BAFF is not associated with much of an increased risk of infections. In contrast, the molecular target of anti-IFNAR1 is a key element of innate and adaptive antiviral immunity, which suggests why anti-IFNAR1 therapy is associated with a higher risk for certain viral infections such as HZ. Long-term studies are needed to evaluate, whether reactivation of other persisting viruses such as CMV, EBV or HPV are associated with related vascular disorders, lymphoma or cervical and epidermal cancers, respectively. Therefore, the use of anti-IFNAR in patients with concomitant risk factors for viral infections including a known primary or secondary immunodeficiency requires caution.

In addition, the ongoing COVID-19 pandemic implies an important need to prime adaptive immunity against a novel virus, a process potentially impaired by all drugs that interfere with the adaptive immune system. How anti-BAFF and anti-IFNAR1 compare in this context is still uncertain but given the important role of type I IFN in antiviral immunity remains important gather more long-term data how anti-IFNAR1 therapy affects the risk for severe COVID-19 severe HZ and its consequences such as post-herpetic neuralgia or other viral infections.

The availability of belimumab and anifrolumab for the treatment of SLE has improved the quality of life of patients with SLE. Individual risk-benefit assessments and shared decision-making are important when considering which therapy is appropriate for which patient.

## Author contributions

All authors researched data, wrote parts of the manuscript, and edited and approved the final version.

## Funding

H-JA received research funding from the Deutsche Forschungsgemeinschaft (AN372/14-4, 20-2, 27-1, 30-1) and from the Volkswagen Foundation (97–744).

## Conflict of interest

H-JA received consultancy or lecture fees from Bayer, GSK, AstraZeneca, Novartis, Otsuka, Janssen, Kezar, Lilly, Sanofi, and PreviPharma. MD’E received consultancy fees from GSK, AstraZeneca, Aurinia, Biogen, and Amgen. KK received consultancy fees from Aurinia. CA has received research support from GSK and consultancy fees from GSK, AstraZeneca, BristolMeyerSqibb and Kezar Inc.

The reviewer JH-Y declared a shared affiliation with the author CA to the handling editor at the time of review.

## Publisher’s note

All claims expressed in this article are solely those of the authors and do not necessarily represent those of their affiliated organizations, or those of the publisher, the editors and the reviewers. Any product that may be evaluated in this article, or claim that may be made by its manufacturer, is not guaranteed or endorsed by the publisher.
